# Allergen Immunotherapy and Possible Clinical Remission: Toward a Disease-Modifying Paradigm in Allergic Disorders

**DOI:** 10.3390/biomedicines14061361

**Published:** 2026-06-17

**Authors:** Fabiana Furci, Remo Poto, Corrado Pelaia, Gilda Varricchi, Chiara Lupia, Vincenzo Patella, Gianenrico Senna, Girolamo Pelaia, Giorgio Walter Canonica

**Affiliations:** 1 Allergy Section, Provincial Healthcare Unit, 89900 Vibo Valentia, Italy; fabianafurci@gmail.com; 2 Department of Translational Medical Sciences, University of Naples Federico II, 80131 Naples, Italy; remo.poto@gmail.com (R.P.); gilda.varricchi@unina.it (G.V.); 3 World Allergy Organization (WAO) Center of Excellence (CoE), 80131 Naples, Italy; 4 Istituti Clinici Scientifici Maugeri-IRCCS Scientific Institute of Telese Terme, 82037 Benevento, Italy; 5 Department of Medical and Surgical Sciences, University “Magna Graecia” of Catanzaro, 88100 Catanzaro, Italy; 6 Center for Basic and Clinical Immunology Research (CISI), University of Naples Federico II, 80131 Naples, Italy; 7 Istituto Endotipi in Oncologia, Metabolismo e Immunologia “G. Salvatore” (IEOMI), National Research Council (CNR), 80131 Naples, Italy; 8 Department of Health Sciences, University “Magna Graecia” of Catanzaro, 88100 Catanzaro, Italy; chiaralupia1996@gmail.com (C.L.); pelaia@unicz.it (G.P.); 9 Department of Internal Medicine ASL Salerno, ‘Santa Maria Della Speranza’ Hospital, 84091 Salerno, Italy; vpatella@tiscali.it; 10 Postgraduate Program in Allergy and Clinical Immunology, University of Naples Federico II, 80131 Naples, Italy; 11 Department of Medicine, University of Verona, 37134 Verona, Italy; gianenrico.senna@univr.it; 12 Allergy Unit, Pederzoli Hospital, 37019 Peschiera, Italy; 13 Department of Biomedical Science, Humanitas University, 20072 Pieve Emanuele, Italy; giorgio_walter.canonica@hunimed.eu; 14 Personalized Medicine, Asthma and Allergy, IRCCS Humanitas Research Hospital, 20089 Rozzano, Italy

**Keywords:** allergen immunotherapy, AIT, asthma, allergic rhinitis, remission

## Abstract

Allergen immunotherapy (AIT) represents the only disease-modifying treatment currently available for IgE-mediated allergic diseases. Traditionally employed to alleviate symptoms and reduce pharmacological dependence, AIT is now being reconsidered within a broader and more ambitious therapeutic framework: the induction of long-term clinical remission. In the field of allergic diseases, the concept of disease control has recently been integrated with that of clinical remission. This review discusses the evolving concept of remission in allergic disorders, particularly allergic rhinitis and allergic asthma, in patients treated with AIT. Starting from the definition of clinical remission, this review aims to analyze the evidence supporting this concept and explore potential tools for clinical application in allergic patients treated with AIT, the only causal therapy.

## 1. Introduction

A new paradigm in the management of allergic diseases has emerged in recent years, posing a conceptual challenge to the traditional definition of clinical remission, which is defined as a state of low or no disease activity and clinical disease control with treatment [1]. This evolving approach promotes a more holistic assessment of therapeutic efficacy and supports a shift from symptom control to disease modification and remission.

The goals of asthma management, according to Global Initiative for Asthma (GINA) guidelines, have shifted from achieving only symptom control to pursuing disease control, preventing and minimizing the risk of future adverse events, such as exacerbations and decline in lung function [2].

In parallel with the transition from the “one size fits all” concept to precision and personalized management, which is based on a deep understanding of the underlying pathophysiological mechanisms and the possibility of using highly effective disease-modifying drugs, the more holistic concept of clinical remission of the disease has become the main goal in asthma management.

There are many definitions currently available for the concept of clinical remission in asthma, apart from the previously cited example, with an increasing need, as reported by international guidelines and consensus, to introduce clinical remission as an outcome in the management of asthma patients and as a goal in clinical trials.

For asthma, clinical remission has been defined as the non-use of systemic oral corticosteroid (OCS), the absence of symptoms, optimization/stabilization of lung function, and patient/provider agreement regarding disease remission [3].

In Italy, the non-use of OCS has been considered the central tenet of “partial” and “complete” clinical remission. Indeed, the Italian Severe Asthma Network Italy (SANI) registry has proposed criteria for asthma remission, classifying patients into two groups: partial and complete remission. In both groups, OCS discontinuation is required together with three major criteria: absence of exacerbations; normal respiratory function, with forced expiratory volume in 1 s (FEV_1_) remaining stable during the year of observation; and asthma control, defined as an Asthma Control Test (ACT) score ≥ 20. When all criteria are met simultaneously, complete remission can be defined. Conversely, when OCS discontinuation and two of the three major criteria are present, partial remission can be considered [4]. It should be emphasized that the SANI definition was developed specifically within a severe asthma registry context. Therefore, although it provides a useful conceptual framework for defining partial and complete remission, it should not be directly extrapolated to patients with mild-to-moderate allergic asthma receiving AIT. In these patients, some criteria, particularly oral corticosteroid withdrawal, may be less informative and should be adapted to disease severity, baseline treatment, and AIT-specific outcomes.

To date, there is no consensus on the criteria to include in the definition of asthma remission. However, in light of the existence of spontaneous remission in asthma patients, of awareness of the chronic inflammatory nature of asthma and of a similar treatment development trajectory in other chronic inflammatory diseases in which the concept of remission is well defined, there is hope that the asthma management paradigm may shift from symptom control to disease modification [5]. In many guidelines, the concept of clinical remission is linked to very mild or no asthma symptoms, no exacerbations and no use of systemic corticosteroids for asthma control [6].

Asthma remission includes spontaneous remission (e.g., during adolescence), remission off treatment (e.g., after allergen immunotherapy, AIT) and remission on treatment (e.g., during treatment with biologics). AIT is the only therapy with proven disease-modifying effects and long-term clinical efficacy for allergic respiratory disorders and may also contribute to clinical remission in severe asthmatic patients treated with biologic therapies. Indeed, although biologics can stabilize the hyperresponsive immune system, restoring immune homeostasis, there is no evidence to date of a disease-modifying effect that lasts after discontinuation. Therefore, AIT remains the only treatment that induces a documented disease-modifying effect [7].

The concept of disease modification is closely linked to that of clinical remission as a general treatment goal in asthma; a notion which has recently been reported in several national guidelines [2].

For allergic rhinitis (AR), studies have reported clinical remission in up to 25% of patients [8,9]. Factors associated with AR remission include the absence of sensitization, older age, and absence of baseline asthma symptoms [10,11]. However, Vasileiadou et al. reported that both sensitization by age 8 and sensitization between ages 8 and 19 years increased the risk of AR incidence and lowered the odds of AR remission [12].

While there is no recognized definition of clinical remission for AR, criteria mentioned by Demoly et al. to evaluate “disease control” in AR can be referred to: measurements of the severity and/or frequency of daily or nocturnal symptoms, impairments in social, physical, professional and educational activities, respiratory function monitoring and exacerbations (e.g., unscheduled medical consultations and rescue medication use) [13]. The European Medicines Agency (EMA) reported four categories to define the effects of AIT on AR in clinical trials: persistent symptoms during AIT, sustained efficacy during the course of AIT, carry-over efficacy after AIT cessation, and cure of allergy. Sustained efficacy during treatment is comparable with “remission on treatment”, whereas carry-over efficacy after AIT cessation and cure of allergy are conceptually closer to “remission off treatment” [14]. Lommatzsch et al. propose an AR remission definition with three components (all must be fulfilled for a period of at least 1 year): minimal or no symptoms (VAS < 2), no exacerbations and no need for systemic steroids for AR treatment [6].

Although the concept of remission is increasingly recognized in chronic airway diseases, its application to allergic respiratory disorders remains challenging because definitions are not yet harmonized and many available studies were not originally designed using remission as a formal endpoint. To improve clarity, the main definitions of remission in allergic diseases are summarized in Table 1.

Within this evolving framework, AIT is of particular interest because, unlike symptomatic pharmacologic treatments, it may induce long-term immunological tolerance and sustained clinical benefit after treatment discontinuation. This makes AIT a relevant candidate for discussion within a remission-oriented therapeutic model.

Focusing on the role of AIT in inducing remission in patients with respiratory allergic disorders, such as AR and asthma, it is important to consider that historically, the evaluation of AIT outcomes was mainly centered on conventional clinical parameters, such as symptom control, reduced medication use, and decreased healthcare resource utilization (e.g., unscheduled visits or emergency department admissions). Conversely, in asthma, the introduction of the concept of clinical remission, already well established in other medical domains such as rheumatology and oncology, is redefining therapeutic goals and may similarly revolutionize the assessment of AIT efficacy [4,15]. In this context, remission refers to the absence of clinically significant symptoms and immunological activity following the discontinuation of therapy [16]. The clinical efficacy of AIT is related to its ability to induce a significant reduction in AR symptoms, decrease the requirement of rescue medications, reduce the risk of developing asthma and maintain its therapeutic effects after discontinuation [17,18,19,20].

This narrative review was based on a literature search conducted in PubMed/MEDLINE, Scopus, and Google Scholar for articles published in English up to 2025. The following keywords and combinations thereof were used: “allergen immunotherapy,” “AIT,” “allergic rhinitis,” “asthma,” “clinical remission,” “disease modification,” “sustained efficacy,” “SCIT,” and “SLIT.” Priority was given to randomized controlled trials, long-term follow-up studies, real-world evidence, position papers, and consensus documents relevant to remission and disease modification in allergic respiratory diseases. Additional references were identified through manual screening of the bibliographies of selected articles.

Based on the available evidence, future AIT trials should consider adopting a pragmatic working definition of AIT-related clinical remission. For asthma, remission should include the absence or minimal presence of symptoms, no clinically relevant exacerbations, no need for systemic corticosteroids, stable or optimized lung function, and no escalation of controller therapy for at least 12 months after AIT discontinuation. For allergic rhinitis, remission should include minimal or no nasal and ocular symptoms, no exacerbations requiring unscheduled medical care, no need for systemic corticosteroids, and minimal or absent rescue medication use for at least 12 months after AIT discontinuation. Across both conditions, patient-reported outcomes, quality of life, and treatment-free sustained benefit should be included as core remission-related outcomes. This proposed framework should be regarded as a practical starting point rather than a validated definition and requires prospective validation in AIT-specific studies.

## 2. Immunological Basis of AIT as a Disease-Modifying Therapy

AIT, administered via subcutaneous or sublingual routes, remains the only available therapeutic option capable of modulating the immune response to achieve long-lasting effects. It is, to date, the sole disease-modifying intervention for IgE-mediated allergic diseases [21]. The therapy possesses a unique capacity to initiate a cascade of immunological events that modulate both innate and adaptive immune responses [22]. The induction of immune tolerance through AIT involves multiple mechanisms, including modulation of allergen-specific memory T and B cell responses, a shift in the balance between allergen-specific IgE and IgG subclasses (notably IgG4), and an increase in the activation threshold of effector cells such as mast cells and basophils [23,24,25]. Furthermore, the immunological mechanisms underlying AIT-induced tolerance share similarities with those observed in other fields of immunology, including autoimmunity, tumor immune evasion, and transplant tolerance [26,27].

Growing evidence suggests that the early introduction of AIT during infancy or early childhood may have the potential to prevent the onset of asthma in children at risk. Several factors contribute to this preventive effect. First, the immune systems of young children are more amenable to modulation, offering a critical window during which a shift toward immune tolerance can be more effectively achieved. Second, the early administration of AIT in children with an allergic predisposition, particularly those presenting with atopic dermatitis and/or food allergy, may interrupt the progression of the atopic march and reduce the likelihood of developing asthma. These findings underscore the importance of early identification of high-risk children and timely initiation of disease-modifying interventions such as AIT [28,29].

Imbalances in immune-cell populations in atopy, namely allergic asthma, and also the restoration of such imbalances using house dust mite (HDM) AIT, have been described for some time [30]. AIT induces long-term remission by modifying immunological memory contained within T cell and/or B cell compartments [31]. AIT inhibits allergen-driven Th2 cell responses, with a consequent decrease in mast cell and basophil recruitment. On the humoral side, AIT induces a class-switch in B cells from IgE to IgG4 and IgA production. Allergen-specific IgG4 and IgA antibodies serve as “blocking antibodies” that compete with IgE for allergen binding, preventing the cross-linking of IgE receptors on effector cells.

A reduction in allergen-specific IgE levels is also observed over time. Concomitantly, levels of IL-4, IL-5, and IL-13 decrease, leading to reduced eosinophil activation and inflammation. Moreover, AIT can restore epithelial barrier integrity and reduce expression of epithelial-derived cytokines such as thymic stromal lymphopoietin (TSLP) and IL-33, which are central to the initiation of allergic responses [32,33,34]. With regard to the marked increases in allergen-specific IgE-blocking activity, IgG4 is reported as the dominant allergen-specific isotype both in serum and local nasal secretions for the subcutaneous (SCIT) route, whereas IgA is reported as the dominant isotype for the sublingual (SLIT) route [32]. IgE-blocking activity was observed to persist at 3 years, 1 year after discontinuation, and these data are important if we remember that long-term “protective” B cell antibody responses may be important for the long-term persistence of tolerance [31].

**Figure 1:**
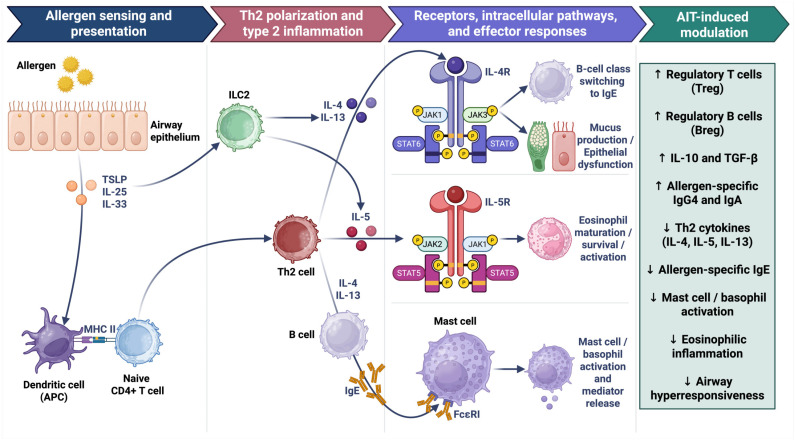
Allergen exposure at the epithelial barrier promotes the release of epithelial-derived alarmins, including TSLP, IL-25, and IL-33, which activate dendritic cells and group 2 innate lymphoid cells (ILC2) and favor Th2 polarization. Th2 cells and ILC2s produce IL-4, IL-5, and IL-13, which sustain type 2 inflammation. IL-4 and IL-13 signal through receptor complexes containing IL-4Rα and activate JAK/STAT6-dependent transcriptional programs involved in IgE class switching, mucus production, epithelial dysfunction, chemokine release, and inflammatory-cell recruitment. IL-5 signaling promotes eosinophil maturation, survival, and tissue persistence. AIT induces immune tolerance by promoting regulatory T cells and regulatory B cells, increasing IL-10 and TGF-β, enhancing allergen-specific IgG4 and IgA blocking antibodies, and reducing effector-cell activation. Created in BioRender. Pelaia, C. (2026) https://BioRender.com/axg4yjo (accessed on 9 June 2026).

The immunological effects of AIT are multifaceted and evolve progressively over the course of treatment, beginning with early-phase desensitization and culminating in long-term immune tolerance. The initial phase is characterized by functional desensitization of effector cells, such as mast cells and basophils, which exhibit a reduced capacity to release histamine and other pro-inflammatory mediators upon allergen exposure. As therapy continues, significant changes in adaptive immunity are observed. AIT promotes the expansion of allergen-specific regulatory Tregs, which secrete immunomodulatory cytokines such as IL-10 and transforming growth factor-beta (TGF-β). These cytokines contribute to the suppression of type 2 inflammation by inhibiting Th2 cell activity and supporting a more tolerogenic immune environment [35]. Type 2 inflammation represents a broader immunological network than the classical adaptive Th2 response, as it involves not only allergen-specific CD4+ Th2 cells but also group 2 innate lymphoid cells, epithelial-derived alarmins, eosinophils, mast cells, basophils, and B cell responses [36]. In allergic airway disease, epithelial barrier disruption and allergen exposure promote the release of thymic stromal lymphopoietin, IL-25, and IL-33, which activate dendritic cells and innate lymphoid cells and amplify Th2 polarization. Activated Th2 cells and ILC2s then produce IL-4, IL-5, and IL-13, which orchestrate the main effector features of type 2 inflammation. At the intracellular level, IL-4 and IL-13 exert many of their biological effects through receptor complexes containing IL-4 receptor alpha subunit (IL-4Rα). IL-4 signals through the type I IL-4 receptor, composed of IL-4Rα and the common gamma chain, whereas both IL-4 and IL-13 can signal through the type II receptor, composed of IL-4Rα and IL-13Rα1. Engagement of these receptor complexes activates Janus kinases and downstream STAT6 signaling, leading to transcriptional programs involved in IgE class switching, mucus hypersecretion, epithelial dysfunction, periostin production, chemokine release, and inflammatory-cell recruitment. In parallel, IL-5 signaling through the IL-5 receptor promotes eosinophil maturation, survival, activation, and tissue persistence, thereby sustaining airway inflammation and remodeling [37]. Therefore, the reduction in IL-4, IL-5, and IL-13 observed during effective AIT should not be interpreted only as a decrease in soluble cytokine concentrations, but as the attenuation of interconnected intracellular pathways that maintain allergic inflammation. AIT may counterbalance these pathways at multiple levels. By increasing allergen-specific regulatory T cells and regulatory B cells, enhancing IL-10 and TGF-β production, and promoting the generation of blocking IgG4 and IgA antibodies, AIT reduces allergen presentation to effector immune cells and progressively increases the threshold for mast-cell and basophil activation [38]. This tolerogenic reprogramming may indirectly downregulate IL-4Rα/JAK/STAT6-driven responses and IL-5-dependent eosinophilic inflammation, thereby linking molecular immune deviation to sustained clinical benefit. In this sense, AIT differs from symptomatic pharmacological treatment because it may reshape the upstream immune response rather than merely suppressing downstream inflammatory mediators (Figure 1).

**Figure 2:**
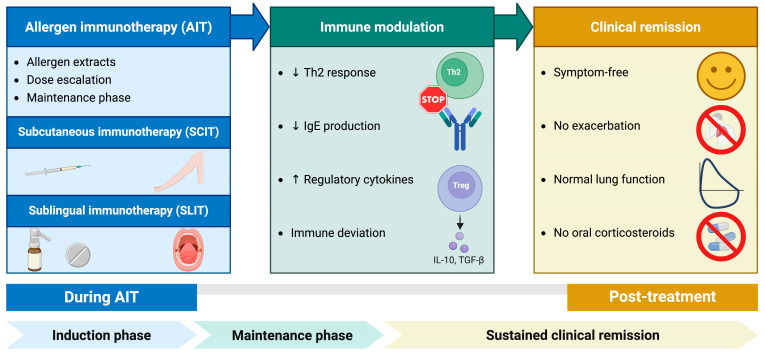
Allergen immunotherapy (AIT) is a disease-modifying strategy leading to clinical remission in allergic disorders. This schematic illustrates the mechanisms and clinical outcomes associated with allergen immunotherapy (AIT). AIT, administered via subcutaneous (SCIT) or sublingual (SLIT) routes, induces progressive immune modulation through multiple coordinated mechanisms. These include the development of immune tolerance, suppression of type 2-mediated responses, reduction in allergen-specific IgE production, and enhancement of regulatory pathways mediated by regulatory T (Treg) cells and immunomodulatory cytokines such as IL-10 and TGF-β. These immunological changes promote a shift toward a more balanced immune profile and decreased effector cell activation. Over time, such effects translate into sustained clinical benefits, including symptom control and, in selected patients, achievement of clinical remission. Clinical remission is characterized by the absence of symptoms and exacerbations, normalization or stabilization of lung function, and reduced or no need for corticosteroid therapy. The lower panel depicts the temporal trajectory of AIT, beginning with an induction phase followed by a maintenance phase, ultimately leading to sustained clinical remission even after treatment discontinuation, highlighting the long-term disease-modifying potential of AIT. This figure is intended to summarize shared disease-modifying mechanisms of AIT and should not be interpreted as implying equivalent remission efficacy between SCIT and SLIT. Created in BioRender. Pelaia, C. (2026) https://BioRender.com/blmua3e (accessed on 9 June 2026).

These complex and coordinated immunological changes underpin the development of immune tolerance, which is a prerequisite for achieving clinical remission, a goal that can be pursued through both SCIT and SLIT (Figure 2).

## 3. Clinical Evidence of Remission Across Allergic Diseases

A critical distinction must be made between studies that explicitly evaluated clinical remission and studies that reported remission-related outcomes without using remission as a predefined endpoint. In the AIT literature, many trials were designed to assess symptom scores, medication use, exacerbation risk, or prevention of asthma onset rather than formal remission. Therefore, the available evidence should be interpreted along a spectrum ranging from direct remission data to indirect indicators of disease modification.

Evidence supporting the remission-related potential of AIT derives largely from studies showing sustained clinical benefit after treatment discontinuation rather than from trials using formal remission as a predefined endpoint [39]. In a placebo-controlled trial evaluating grass pollen SCIT, the authors compared seasonal symptom scores between patients who discontinued immunotherapy after 3 years and those who continued treatment for 6 to 7 years. No significant differences were observed between these groups, suggesting that a 3-year course of AIT may be sufficient to achieve long-term clinical benefit. Importantly, the sustained reduction in seasonal symptoms and the decreased need for rescue medication were also associated with persistent improvements in health-related quality of life. From an immunological standpoint, this long-term benefit was accompanied by the suppression of late-phase allergic responses, characterized by a reduction in CD4+ T cells and decreased expression of mRNA encoding type 2 inflammation cytokines [40].

Additional evidence for the long-term efficacy of AIT comes from randomized, double-blind, placebo-controlled studies. In a trial evaluating the effects of a 3-year course of ragweed SCIT, persistent clinical and immunological benefits were observed 1 year after treatment discontinuation. Notably, suppression of inflammatory mediators in nasal secretions remained detectable following blinded withdrawal of immunotherapy [41]. Passalacqua et al., comparing patients with mite-induced rhinoconjunctivitis treated with SLIT with the placebo group, highlighted a significant reduction in inflammatory-cell infiltration after conjunctival challenge, and ICAM-1 expression on conjunctival epithelium in the first year of treatment in the immunotherapy group. These effects were also reported for the minimum persistent inflammation in symptom-free patients exposed constantly to allergens. Moreover, a significant reduction in serum concentrations of eosinophil cationic protein was also seen [42].

In a prospective, open, controlled 15-year follow-up study, Marogna et al. evaluated patients with AR and mite-induced bronchial hyperreactivity, with or without asthma, who were divided into four groups receiving pharmacotherapy alone or SLIT for 3, 4, or 5 years. The authors performed yearly assessments, and a clinical diary card was used to record the symptom-plus-medication score (SMS) from September to February. The clinical efficacy of SLIT was arbitrarily considered to persist as long as the SMS remained <50% of the baseline value. In light of the results reported in this study, the authors emphasized a distinctive property of SLIT not shared by pharmacologic treatments: long-lasting efficacy after discontinuation and modification of the natural course of allergic disease, with clinical and economic relevance. In particular, this study reported that the long-term effect (>4 years) of SLIT correlates with the duration of immunotherapy and that 4 years appears to be the optimal duration for inducing a long-lasting effect. A duration of 5 years provided only marginal additional benefits [43]. These clinical results are in agreement with other studies concerning SLIT and SCIT [24,44,45]. However, these studies were not designed to evaluate remission according to standardized criteria, and therefore they should be interpreted as supporting sustained efficacy and disease modification rather than directly demonstrating clinical remission.

SCIT demonstrated the persistence of subjective symptom improvement 4 years after cessation of immunotherapy, observed both in actively treated patients and to a lesser extent in the placebo group [46]. Comparable findings have been reported for SLIT, with multiple studies documenting long-term remission of allergic symptoms in both adults and children. These results further support the disease-modifying potential of AIT, irrespective of the administration route, and its capacity to induce sustained immunological and clinical benefits beyond the treatment period [47,48,49]. Moreover, considering that the effect of SLIT on bronchial hyperresponsiveness has been reported in studies conducted both in children and adults, it is plausible that the result on PD20 reflects the anti-inflammatory effect of AIT, although inflammation is only one of the components of bronchial hyperreactivity.

The relative efficacy of SCIT and SLIT in achieving sustained remission off treatment remains difficult to compare directly. To date, most studies evaluating long-term outcomes after AIT were not designed as head-to-head comparisons between routes and did not use standardized remission criteria as predefined endpoints. Evidence supporting SCIT includes long-term follow-up studies showing persistent clinical benefit after treatment discontinuation, whereas evidence supporting SLIT includes randomized trials and real-world studies demonstrating sustained reductions in symptoms, medication use, asthma risk, and disease progression. However, differences in allergen extracts, treatment duration, adherence, age groups, baseline disease severity, and follow-up length limit indirect comparisons. Therefore, the available evidence supports the disease-modifying potential of both SCIT and SLIT but does not establish the superiority of one route over the other for inducing formal clinical remission. In clinical practice, the choice between SCIT and SLIT should be individualized according to allergen availability, product-specific evidence, safety profile, adherence, patient preference, and feasibility.

Bearing in mind that AIT is considered a causal therapy, long-term follow-up data play a key role. In a recent paper, Harintajinda et al. evaluated long-term efficacy and aimed to identify predictive factors in the clinical remission of AR patients who had completed a three-year course of house dust mite subcutaneous immunotherapy (HDM SCIT) and then discontinued it, considering the no-further-need or use of daily intranasal steroid or oral antihistamine as clinical remission. They reported that HDM SCIT shows long-term efficacy after treatment discontinuation. In particular, the authors reported that AR remission can be maintained in 79% of patients after discontinuation for 5 years. Moreover, the authors highlighted that starting HDM SCIT before the age of 15 and the absence of asthma are significantly correlated with AR remission. This finding should be interpreted as sustained treatment-free control according to the authors’ predefined criteria, rather than as complete remission defined by the total absence of symptoms and all medications [50].

Two large randomized controlled trials (RCTs) with SQ HDM SLIT tablets have confirmed the efficacy of AIT in asthma patients. One study reported a moderate statistically significant reduction in daily inhaled corticosteroid (ICS) dose required to maintain asthma control compared to placebo, while another RCT reported that the addition of HDM SLIT in asthma patients, not well controlled by ICS, improved time to first moderate or severe asthma exacerbation during ICS step-down [51,52].

The REACT study provides important real-world evidence for the long-term effectiveness of AIT, including reductions in medication use, improved asthma control, fewer exacerbations, and fewer respiratory tract infections [53]. However, formal clinical remission was not assessed as a primary outcome, and the study should therefore be interpreted as supporting disease modification rather than directly documenting remission. In particular, regarding asthma, in patients treated with AIT for AR and pre-existing asthma, a significant reduction in both controller and reliever medication was observed, highlighting improved asthma control. Moreover, in relation to asthma treatment step-down, AIT reduced the odds of experiencing asthma exacerbations, with a relevant role in the prevention of asthma progression [53]. These results are related to some evidence reporting that HDM SLIT has been shown to restore impaired innate antiviral immunity [54,55]. A less severe asthmatic population, as only 4% were at treatment step 4, supported the hypothesis that AIT plays a key role in preventing progression from mild to severe asthma and should be prescribed earlier in the treatment of allergic asthmatic patients [56]. Moreover, Woehlk et al. reported that patients treated with AIT were less likely to experience respiratory tract infections, which are known to be risk factors for asthma exacerbations, requiring antibiotics. Data regarding the clinical benefits of AIT were supported by reductions in hospitalizations, length of stay and related costs in AR and asthma outcomes [57].

Durham et al. reported that in patients with seasonal grass pollen-induced AR, with or without concomitant seasonal asthma, a reduction in the mean rhinoconjunctivitis daily symptom score by 25% to 36% in patients treated with SQ-standardized sublingual grass immunotherapy compared with the placebo group over the five grass pollen seasons (three of treatment and two of follow-up) covered by the trial was observed [58]. The rhinoconjunctivitis medication score was reduced by 20% to 45% in favor of active treatment. This clinical improvement was accompanied by sustained immunological effects, including a persistent increase in allergen-specific IgG4 levels, which remained elevated for up to 2 years following discontinuation of SLIT. This sustained IgG4 increase was accompanied by parallel increases in serum IgE-blocking factor and in serum inhibitory activity for the binding of allergen-IgE complexes to B cells. In a long-term, double-blind, randomized study conducted in Japan, the efficacy and optimal dosing of a fast-dissolving sublingual tablet for Japanese cedar (*Cryptomeria japonica*) pollen allergy were both evaluated in adults and adolescents with moderate to severe seasonal allergic rhinoconjunctivitis. During the first year of treatment, a daily dose of 5000 Japanese Allergy Units (JAU) was identified as optimal. Notably, a linear trend of continued clinical improvement was observed over 3 years of treatment, compared with outcomes assessed at 18 months. The study also demonstrated a 30–40% reduction in combined symptom and medication scores compared to placebo, with this clinical benefit persisting for up to 2 years following cessation of SLIT. These findings support both the long-term efficacy and the sustained disease-modifying effect of SLIT in Japanese cedar pollen allergy [49].

The Preventive Allergy Treatment (PAT) study provided pivotal evidence supporting the long-term preventive effects of AIT. In this randomized trial, children with grass pollen allergy who received SCIT exhibited a significantly lower risk of developing asthma over a 10-year follow-up period compared to those receiving standard pharmacotherapy [59]. Although these findings are relevant to the concept of disease modification, they do not constitute direct evidence of clinical remission, since remission was not used as a predefined endpoint.

The Grass Sublingual Immunotherapy Tablet Asthma Prevention (GAP) trial evaluated the effect of the SQ grass sublingual immunotherapy tablet compared to placebo regarding the risk of developing asthma. The study enrolled 812 children with seasonal allergic rhinoconjunctivitis, but no prior diagnosis of asthma, who were randomized to receive a fast-dissolving grass pollen SLIT tablet for three years, followed by a two-year blinded observational follow-up. The trial demonstrated a statistically significant reduction in the risk of developing asthma symptoms or requiring asthma medication during the follow-up period. Moreover, when including FEV_1_ reversibility ≥ 12%, the proportion of patients fulfilling the asthma criteria was lower among those treated with SLIT [60].

In a randomized clinical trial on 834 adults with HDM allergy-related asthma not well controlled by ICS or combination products, and with HDM allergy-related rhinitis, in which the primary outcome was time to first moderate or severe asthma exacerbation during the ICS reduction period (when ICS was reduced by 50% for 3 months and then completely withdrawn for 3 months), Virchow et al., evaluated the efficacy and adverse events of the HDM SLIT tablet vs. placebo. From this analysis, the authors reported that the addition of HDM SLIT to maintenance medications improved time to first moderate or severe asthma exacerbation during ICS reduction compared with placebo, with an estimated absolute reduction at 6 months of 9 to 10 percentage points. Therefore, the fact that the HDM SLIT tablet plays a preventative role in asthma exacerbations when ICS was reduced could indicate an anti-inflammatory action of the active treatment that can maintain the level of asthma control even in the absence of ICS. Efficacy was assessed in all asthma exacerbations, in general, as opposed to exacerbations only induced by HDM exposure [52].

In the EfficAPSI real-world study (RWS), evaluating the real-world impact of personalized SLIT-liquid on the prevention of asthma onset or worsening in treated AR patients, with or without asthma, the authors highlighted that SLIT-liquid exposure was associated with a significantly lower risk of asthma onset (36%) compared with patients using symptomatic AR medications alone (controls), which was consistently observed for both male and female patients and for the different allergen subgroups analyzed (HDM, grass, birch and ragweed), and a reduced risk of asthma worsening as demonstrated by a lower incidence of asthma treatment step-up for the SLIT group, regardless of baseline treatment step [61]. In particular, in the evaluation of the effect on the progression of asthma in patients with pre-existing asthma at the index date, GINA recommendations were followed to determine the highest treatment step for each patient in each year. The authors found an increased probability of GINA step-down, about 50%, depending on GINA baseline step and year of follow-up [61].

In the BREATH studies, a reduction in progression of asthma was reported in about 20–40% in patients treated with SLIT (liquid or tablets) and in a similar range with birch pollen SCIT (natural or modified), compared with patients treated with symptomatic treatments alone [62,63,64,65].

Overall, only a limited number of studies have explicitly assessed remission as a clinical endpoint after AIT, whereas most available trials report remission-related outcomes indirectly through sustained symptom control, medication reduction, exacerbation prevention, or delayed disease progression. This distinction is important because the current evidence base is more robust for long-term efficacy and disease modification than for formal remission defined according to standardized criteria. Accordingly, the concept of remission after AIT remains promising, but still insufficiently standardized across studies and allergic disease settings.

Although the available literature supports the disease-modifying potential of AIT, the overall evidence base remains heterogeneous and should be interpreted with caution. Studies differ substantially in terms of patient age, baseline disease severity, allergen sensitization pattern, presence of comorbid asthma or rhinitis, and previous treatments. In addition, interventions are not uniform, as trials evaluate different allergen extracts, formulations, routes of administration, treatment durations, and follow-up periods. Outcomes also vary widely and frequently include symptom scores, medication reduction, exacerbation risk, or asthma onset, whereas formal clinical remission is rarely a prespecified endpoint. Several pivotal studies have important limitations. Prevention trials such as PAT and GAP were conducted in selected pediatric populations and therefore may not be fully generalizable to broader patient groups, including adults or patients with more severe disease. Although these real-world studies provide valuable information on long-term effectiveness in routine clinical practice, their findings should be interpreted in light of the methodological limitations inherent to retrospective claims-based analyses. In particular, studies such as REACT, EfficAPSI, and the BREATH database analyses may be affected by confounding by indication, selection bias, residual confounding, diagnostic coding inaccuracies, and incomplete information on symptom burden, lung function, adherence to AIT or background medication, allergen exposure, and patient-reported outcomes. Moreover, medication dispensing or prescription data do not necessarily reflect actual treatment use. Therefore, these studies strongly support the long-term effectiveness and disease-modifying potential of AIT, but they cannot by themselves establish clinical remission according to harmonized and prospectively defined criteria.

An additional limitation is the potential for publication bias. Studies reporting positive or sustained AIT-related outcomes may be more likely to be published, whereas negative, null, or inconclusive remission-related findings may be underrepresented in the literature. This could lead to an overestimation of the apparent ability of AIT to induce sustained clinical remission, particularly because formal remission endpoints have rarely been prospectively defined. Future clinical trials and real-world registries should therefore report negative and neutral outcomes as systematically as positive findings. Pediatric remission after AIT deserves specific consideration. Adult remission criteria, particularly those centered on oral corticosteroid withdrawal or stabilization of established lung-function impairment, may not be fully applicable to children, especially when AIT is used before asthma has developed. In pediatric populations, remission-related outcomes should include age-appropriate symptom control, absence of exacerbations, reduced need for rescue or controller medication, preservation of normal lung-function trajectories, and prevention of asthma onset or worsening. Additional child-specific outcomes may include school attendance, sleep quality, physical activity, normal growth, treatment burden, and quality of life. Prevention of new clinically relevant sensitizations and interruption of the atopic march may also be considered exploratory disease-modifying endpoints. Therefore, in children, AIT-related remission should be interpreted not only as the disappearance of established disease activity, but also as prevention of progression toward persistent or more severe allergic respiratory disease.

Another important limitation is that not all patients treated with AIT achieve sustained remission. Clinical non-response may reflect inadequate adherence, insufficient treatment duration, allergen mismatch, high polysensitization burden, advanced airway remodeling, severe or uncontrolled disease, or persistent environmental exposure. Moreover, the lack of validated predictive biomarkers makes it difficult to identify, before treatment initiation, which patients are most likely to achieve long-term benefit. For these reasons, the concept of remission after AIT remains promising but should still be regarded as an evolving therapeutic target rather than a uniformly achievable outcome.

The principal clinical trials and real-world studies evaluating remission-related or disease-modifying outcomes of allergen immunotherapy in this review are summarized in Table 2.

## 4. Molecular Signatures During AIT: Unmet Needs to Obtain Long-Term Outcomes

From the perspective of a personalized immunological approach to AIT, there is a growing need for reliable clinical biomarkers to identify responders, guide patient selection, and optimize therapeutic strategies. Biomarkers could play a pivotal role in guiding treatment indication, monitoring immune response, and tailoring the duration and intensity of therapy. Traditionally, AIT has been contraindicated in unstable and/or severe asthma, but with the availability of biologics, AIT could be reconsidered in these patients, inducing sustained remission in severe asthma, but highlighting that it should be started only once asthma is fully controlled without symptoms or risk factors for exacerbations. A European Academy of Allergy and Clinical Immunology Position Paper has reported the need for a universally harmonized approach to clinical outcome measures of AIT in asthma [66]. Moreover, for biomarkers related to AIT in asthma patients, the challenge is even greater, as the commonly used biomarkers, such as fractional exhaled nitric oxide (FeNO), serum eosinophil count, and sputum eosinophils, are influenced by biologic therapies. Therefore, focus could be put on additional outcome measures such as local symptoms and quality of life (QoL) [66].

Several challenges hinder progress in this area, including the lack of standardization in biomarker assays, limited reproducibility of results across different laboratories, heterogeneity in the definition of responders and non-responders, and technical limitations associated with immunological analyses. Overcoming these barriers is essential for the integration of biomarker-driven approaches into routine clinical management of allergic diseases treated with AIT [31]. The identification of several immunological parameters, such as reductions in allergen-specific IgE, increases in IgG4 levels, and modulation of basophil activation test (BAT) responses has been associated with favorable clinical outcomes. However, these biomarkers lack sufficient specificity and, to date, have proven inadequate for reliably predicting sustained unresponsiveness after treatment discontinuation.

Component-resolved diagnostics (CRD) may enhance patient stratification, particularly in polysensitized individuals, by distinguishing sensitization to major versus minor allergenic components and thereby guiding more precise therapeutic choices [67]. Moreover, emerging evidence highlights the importance of regulatory immune cell subsets, namely, Tregs and regulatory B cells (Bregs), as potential correlates of immunological tolerance. Advances in high-throughput ‘omics’ technologies, including transcriptomics, proteomics, and metabolomics, are being employed to identify molecular signatures that secure long-term outcomes during AIT. Nonetheless, these approaches remain at the investigational stage, and robust clinical validation is still required before they can be integrated into routine clinical practice [68].

## 5. Challenges and Knowledge Gaps

Despite promising findings, several barriers hinder the widespread implementation of remission as a therapeutic goal. First, the lack of consensus on the definition of remission across allergic diseases complicates study design and interpretation. In both allergic rhinitis and asthma patients treated with AIT, complete and long-term remission remains a conceptual framework requiring harmonization. Second, the absence of validated biomarkers prevents early identification of potential “remitters.” Third, treatment adherence remains suboptimal due to the prolonged nature of AIT and the burden of administration, particularly in pediatric populations.

A balanced interpretation of AIT outcomes requires acknowledgment that a proportion of patients do not achieve complete or sustained remission. Some patients experience only partial benefit, while others discontinue treatment because of inconvenience, adverse effects, or poor adherence. Possible explanations for suboptimal response include inaccurate patient selection, polysensitization with clinically relevant allergens not fully covered by treatment, persistent exposure to high allergen loads, severe disease with established tissue remodeling, and interindividual variability in immunological responsiveness. These observations reinforce the need for better phenotyping and biomarker-guided selection of candidates for AIT.

Economic considerations and access disparities further limit the use of AIT, especially in low- and middle-income countries. Additionally, most available data derive from monosensitized patients, whereas polysensitization is increasingly prevalent. Finally, the integration of AIT with emerging therapies such as biologics requires further exploration in well-controlled trials.

## 6. Future Directions and Clinical Implications

The future of AIT lies in its evolution from a symptom-reducing to a remission-inducing therapy. To realize this potential, a number of priorities must be addressed. First, international consensus is needed on the definition and metrics of remission in allergic diseases. Second, prospective cohort studies and randomized controlled trials should be designed with remission as a primary or secondary endpoint.

Third, the identification and integration of predictive biomarkers of clinical response could allow monitoring of desensitization, efficacy, and the likelihood of response, contributing to accelerating personalized medicine and improving patient care [69]. Fourth, combination strategies using AIT with biologics—such as anti-IgE, anti-IL-4R, or anti-TSLP agents—may improve remission rates, particularly in difficult-to-treat cases. Finally, health technology innovations, including digital adherence tools and remote monitoring, may enhance the efficiency and accessibility of AIT programs.

## 7. AIT and Biologics: A Potential Window of Opportunity for Tolerance Induction

The integration of AIT with biologic therapies represents one of the most relevant future directions in the management of allergic respiratory diseases, particularly in patients with moderate-to-severe asthma or complex type 2 inflammatory phenotypes. Traditionally, uncontrolled or severe asthma has been considered a major limitation to the initiation of AIT because of safety concerns and the risk of exacerbation. However, the availability of biologics targeting key type 2 inflammatory pathways may modify this scenario by improving disease stability and creating a more favorable immunological environment for AIT [70]. From a mechanistic perspective, biologics may reduce the acute inflammatory burden that interferes with tolerance induction. Anti-IgE therapy may decrease free IgE levels and FcεRI expression on mast cells and basophils, thereby reducing effector-cell activation during allergen exposure. Anti-IL-4Rα therapy blocks signaling induced by both IL-4 and IL-13, attenuating STAT6-dependent pathways involved in IgE class switching, mucus production, epithelial activation, and chemokine release. Anti-IL-5 or anti-IL-5 receptor strategies reduce eosinophilic inflammation, whereas anti-TSLP therapy acts upstream by targeting an epithelial alarmin involved in the initiation and amplification of type 2 inflammation [71]. By suppressing these inflammatory pathways, biologics could theoretically create a “window of opportunity” in which AIT may more effectively promote allergen-specific Treg- and Breg-mediated tolerance. This concept is particularly relevant because biologics and AIT may have complementary therapeutic profiles. Biologics provide powerful control of active type 2 inflammation, but their disease-modifying effect after discontinuation remains uncertain. Conversely, AIT has the potential to induce persistent allergen-specific immune tolerance, but its use may be limited in patients with uncontrolled disease. A combined or sequential strategy may therefore be rational: biologics could first stabilize the inflammatory milieu and reduce clinical risk, while AIT could subsequently promote long-term immune reprogramming and potentially contribute to remission off treatment [72].

From a clinical perspective, biologics should currently be viewed primarily as a potential stabilizing or bridging strategy in selected patients whose asthma is insufficiently controlled to allow safe initiation of AIT. In such cases, biologic treatment may first reduce exacerbation risk and improve disease stability, after which AIT could be considered to promote allergen-specific immune tolerance. Concomitant biologic–AIT treatment may also be considered in selected difficult-to-treat patients, but this approach should not be generalized because robust prospective evidence is still limited. The optimal duration of biologic pre-treatment before AIT initiation has not been established and should be individualized according to asthma control, exacerbation history, lung function, and overall risk profile. Safety issues should also be explicitly considered, including systemic reactions to AIT, the need for supervised administration, and careful monitoring when treatment is initiated or escalated. Although anti-IgE therapy has been proposed as a potential strategy to improve tolerability in selected patients, the combination of AIT and biologics remains an evolving precision-medicine approach that requires dedicated controlled studies.

Nevertheless, this approach remains largely hypothesis-generating. Several key questions remain unresolved, including which biologic should be preferred for specific allergic phenotypes, whether biologics should be used before, during, or after AIT, how long combination treatment should be continued, and which biomarkers should be used to monitor tolerance induction. Prospective controlled studies are needed to determine whether combined AIT-biologic strategies can increase remission rates, improve safety, shorten the time required to achieve immune tolerance, or prolong clinical benefit after treatment discontinuation. Until such data are available, the combination of AIT and biologics should be considered a promising but still evolving strategy within precision medicine.

## 8. Conclusions

Remission is an increasingly attainable and clinically meaningful objective in the management of allergic diseases, such as AR and asthma. AIT, by exerting profound modulatory effects on both innate and adaptive immunity, has the potential to induce long-term clinical and immunological remission in selected patients.

To fully exploit the therapeutic potential of AIT in the era of precision medicine, continued efforts are needed to refine operational definitions of clinical remission, identify and validate predictive biomarkers, and implement individualized treatment strategies. These advances will be instrumental in optimizing patient selection, maximizing long-term benefits, and minimizing unnecessary treatment exposure.

## Figures and Tables

**Table 1:** Comparison of definitions and proposed criteria for clinical remission or disease control in asthma and allergic rhinitis.

Disease	Source/Framework	Concept Addressed	Main Criteria/Components	Main Strengths	Main Limitations
Asthma	GINA-based management framework [2]	Disease control/remission-oriented treatment target	Mild or absent symptoms, no exacerbations, minimization of future risk, stable or optimized lung function, no need for systemic corticosteroids.	Widely recognized clinical framework; useful for routine practice.	Focuses primarily on control rather than on a standardized definition of remission.
Asthma	Menzies-Gow et al. [15]	Clinical remission in asthma	Absence of symptoms, no exacerbations, no maintenance oral corticosteroids, optimization or stabilization of lung function.	Helps operationalize remission as a multidimensional concept.	Not universally adopted; some components remain partly subjective.
Asthma	SANI criteria [4]	Partial and complete clinical remission in severe asthma	Oral corticosteroid withdrawal, absence of exacerbations, normal or stable respiratory function, and asthma control assessed by Asthma Control Test (ACT) score ≥ 20.	Practical and clinically applicable; provides partial and complete remission categories.	Developed within a national registry context; not yet internationally harmonized.
Allergic rhinitis	Demoly et al. [13]	Disease control in allergic rhinitis	Severity and frequency of daily and nocturnal symptoms, impact on social, physical, professional, and educational activities, respiratory function monitoring, exacerbations, unscheduled visits, and rescue medication use.	Useful for multidimensional assessment of rhinitis burden.	Refers to disease control, not a formal remission definition.
Allergic rhinitis	EMA categories for AIT trials [14]	Clinical effect categories relevant to remission concepts	Persistent symptoms during AIT; sustained efficacy during AIT; carry-over efficacy after AIT cessation; cure of allergy.	Useful for interpreting AIT outcomes over time.	Developed for regulatory trial evaluation, not as a patient-level clinical remission definition.
Allergic rhinitis	Lommatzsch et al. [6]	Proposed clinical remission in allergic rhinitis	All 3 required: minimal or no symptoms (VAS < 2), no exacerbations, and no need for systemic steroids for allergic rhinitis treatment.	Clear and easy-to-apply proposal; introduces a remission-oriented framework for allergic rhinitis.	Not yet validated or broadly standardized; less comprehensive than asthma remission proposals.

**Table 2:** Main clinical trials and real-world studies evaluating remission-related or disease-modifying outcomes of allergen immunotherapy.

Study	Design	Population	Allergen/Disease	AIT Type/Duration	Follow-Up	Key Remission- or Disease-Modifying Outcome
Durham et al., 2012 [58]	Randomized trial with post-treatment follow-up	Patients with seasonal grass pollen-induced allergic rhinitis, with or without seasonal asthma	Grass pollen allergic rhinoconjunctivitis	SQ-standardized SLIT; 3 years	2 years after cessation	Clinical improvement and persistent immunological effects remained after discontinuation.
Mosbech et al., 2014 [51]	Randomized double-blind placebo-controlled trial	Adults with HDM-allergic asthma controlled on inhaled corticosteroids	HDM allergic asthma	SLIT tablet; treatment duration per trial protocol	During treatment period	Reduced inhaled corticosteroid dose while maintaining asthma control.
Virchow et al., 2016 [52]	Randomized clinical trial	834 adults with HDM-related asthma not well controlled by ICS or combination therapy, with HDM allergic rhinitis	HDM allergic asthma	SLIT tablet; add-on treatment during ICS reduction	6 months during step-down period	Improved time to first moderate or severe asthma exacerbation during ICS reduction.
GAP trial (Valovirta et al., 2018) [60]	Randomized placebo-controlled trial	812 children with seasonal allergic rhinoconjunctivitis and no prior asthma diagnosis	Grass pollen allergy	SLIT tablet; 3 years	2-year blinded follow-up (5 years total)	Reduced risk of asthma symptoms or asthma medication use during follow-up.
BREATH studies (2017–2019) [62,63,64,65]	Retrospective real-world database analyses	Patients with allergic rhinitis and/or asthma	Grass or birch pollen respiratory allergy	SLIT tablets/liquid or birch SCIT; routine care	Long-term database follow-up	Reduced progression toward asthma in AIT-treated patients compared with symptomatic treatment alone.
Yonekura et al., 2021 [49]	Long-term double-blind randomized study	Adults and adolescents with moderate-to-severe Japanese cedar pollen allergic rhinoconjunctivitis	Japanese cedar pollen allergy	SLIT tablet; 3 years	2 years after cessation	30–40% reduction in combined symptom-medication scores versus placebo.
REACT study (Fritzsching et al., 2021) [53]	Retrospective claims-based real-world cohort	Patients with allergic rhinitis, including those with pre-existing asthma	Mixed respiratory allergy in routine care	AIT in routine practice; modality varies	Long-term follow-up (claims data 2007–2017)	Associated with reduced allergic rhinitis and asthma medication use, fewer exacerbations, and fewer respiratory tract infections.
Harintajinda et al., 2025 [50]	Long-term follow-up study	Patients completing a 3-year HDM SCIT course	HDM allergic rhinitis	SCIT; 3 years	5 years after discontinuation	Sustained treatment-free control was maintained in a large proportion of patients according to predefined criteria.

## Data Availability

No new data were created or analyzed in this study.
